# Supplementary material for: COVID-19 and Adverse Pregnancy Outcome: A Systematic Review of 104 Cases
Source: J Clin Med. 2020 Oct 26;9(11):3441. doi: 10.3390/jcm9113441 (PMC7692613; doi:10.3390/jcm9113441)
Supplement: Supplementary file 1 [file jcm-09-03441-s001.pdf]

Supplementary Materials

COVID-19 and Adverse Pregnancy Outcome: A Systematic Review of 104 Cases

Table of contents

1. PRISMA Checklist 1
2. Search strategy 3
3. Reasons for study exclusion....................4
4. Detailed distribution of patients on the studies included studies....................5
5. References 11

1. PRISMA Checklist

Table S1. Checklist summarizing compliance with PRISMA guidelines*.

Section/topic	#	Checklist item	Reported on page #
**TITLE**			
Title	1	Identify the report as a systematic review, meta-analysis, or both.	Title
ABSTRACT			
Structured summary	2	Provide a structured summary including, as applicable: background; objectives; data sources; study eligibility criteria, participants, and interventions; study appraisal and synthesis methods; results; limitations; conclusions and implications of key findings; systematic review registration number.	1
INTRODUCTION			
Rationale	3	Describe the rationale for the review in the context of what is already known.	2
Objectives	4	Provide an explicit statement of questions being addressed with reference to participants, interventions, comparisons, outcomes, and study design (PICOS).	2
METHODS			
Protocol and registration	5	Indicate if a review protocol exists, if and where it can be accessed (e.g., Web address), and, if available, provide registration information including registration number.	N/A
Eligibility criteria	6	Specify study characteristics (e.g., PICOS, length of follow-up) and report characteristics (e.g., years considered, language, publication status) used as criteria for eligibility, giving rationale.	3
Information sources	7	Describe all information sources (e.g., databases with dates of coverage, contact with study authors to identify additional studies) in the search and date last searched.	3
Search	8	Present full electronic search strategy for at least one database, including any limits used, such that it could be repeated.	3
Study selection	9	State the process for selecting studies (i.e., screening, eligibility, included in systematic review, and, if applicable, included in the meta-analysis).	3 (Figure1)
Data collection process	10	Describe method of data extraction from reports (e.g., piloted forms, independently, in duplicate) and any processes for obtaining and confirming data from investigators.	3
Data items	11	List and define all variables for which data were sought (e.g., PICOS, funding sources) and any assumptions and simplifications made.	3
Risk of bias in individual studies	12	Describe methods used for assessing risk of bias of individual studies (including specification of whether this was done at the study or outcome level), and how this information is to be used in any data synthesis.	N/A
Summary measures	13	State the principal summary measures (e.g., risk ratio, difference in means).	3

Synthesis of results	14	Describe the methods of handling data and combining results of studies, if done, including measures of consistency (e.g., I^2) for each meta-analysis.	4
Risk of bias across studies	15	Specify any assessment of risk of bias that may affect the cumulative evidence (e.g., publication bias, selective reporting within studies).	N/A
Additional analyses	16	Describe methods of additional analyses (e.g., sensitivity or subgroup analyses, meta-regression), if done, indicating which were pre-specified.	N/A
RESULTS			
Study selection	17	Give numbers of studies screened, assessed for eligibility, and included in the review, with reasons for exclusions at each stage, ideally with a flow diagram.	4-9
Study characteristics	18	For each study, present characteristics for which data were extracted (e.g., study size, PICOS, follow-up period) and provide the citations.	4-9, Table 1, Supplementary tables
Risk of bias within studies	19	Present data on risk of bias of each study and, if available, any outcome level assessment (see item 12).	Supplementary tables
Results of individual studies	20	For all outcomes considered (benefits or harms), present, for each study: (a) simple summary data for each intervention group (b) effect estimates and confidence intervals, ideally with a forest plot.	4-12, Supplementary tables
Synthesis of results	21	Present results of each meta-analysis done, including confidence intervals and measures of consistency.	4-12
Risk of bias across studies	22	Present results of any assessment of risk of bias across studies (see Item 15).	N/A
Additional analysis	23	Give results of additional analyses, if done (e.g., sensitivity or subgroup analyses, meta-regression [see Item 16]).	N/A
DISCUSSION			
Summary of evidence	24	Summarize the main findings including the strength of evidence for each main outcome; consider their relevance to key groups (e.g., healthcare providers, users, and policy makers).	12-13
Limitations	25	Discuss limitations at study and outcome level (e.g., risk of bias), and at review-level (e.g., incomplete retrieval of identified research, reporting bias).	13
Conclusions	26	Provide a general interpretation of the results in the context of other evidence, and implications for future research.	13
FUNDING			
Funding	27	Describe sources of funding for the systematic review and other support (e.g., supply of data); role of funders for the systematic review.	13

*Moher D, Liberati A, Tetzlaff J, Altman DG; PRISMA Group (2009) Preferred reporting items for systematic reviews and meta-analyses: the PRISMA statement. J Clin Epidemiol 62:1006-1012.

2. Search strategy

We searched PubMed, MEDLINE, Scopus, Embase and Web of Science for reports published only in English until May 4, 2020, that assessed all informations of pregnant patients with COVID-19. We searched all fields for coronavirus (search terms:"Coronavirus 19", "COVID-19", "SARS-CoV-2", "2019-nCoV", "novel coronavirus 2019") and terms of pregnancy (search terms:"pregnan*").

Full search strategies for each database are given in Table S2.

Table S2. Search strategy.

Database	Number of studies	Search terms
**PubMed**	172	("Coronavirus 19" OR "COVID-19" OR "SARS-CoV-2" OR "2019-nCoV" OR "novel coronavirus 2019")<br>AND<br>("pregnan*")
**Medline**	58	("Coronavirus 19" OR "COVID-19" OR "SARS-CoV-2" OR "2019-nCoV" OR "novel coronavirus 2019")<br>AND<br>("pregnan*")
**Scopus**	34	TITLE-ABS-KEY("Coronavirus 19" OR "COVID-19" OR "SARS-CoV-2" OR "2019-nCoV" OR "novel coronavirus 2019)<br>AND TITLE-ABS-KEY("pregnan*")
**Embase**	45	('Coronavirus 19' OR 'COVID-19' OR 'SARS-CoV-2' OR '2019-nCoV' OR 'novel coronavirus 2019')<br>AND<br>('pregnan*')
**Web of Science**	12	((TS=Coronavirus 19) OR (TS=COVID-19) OR (TS=SARS-CoV-2) OR (TS=2019-nCoV) OR (TS=novel coronavirus 2019))<br>AND<br>((TS=pregnan*))

3. Reasons for study exclusion

We manually screened the retrieved articles which were met inclusion criteria. After excluding studies by examining titles and abstracts, full texts of 11 case series articles were eligible for inclusion.

88 studies were retrieved following reasons**:**

Table S3. Reason for exclusion during full text screening.

Number of studies	Reason
**27**	Articles without case series
**25**	Focusing on treatment or management, not on prognosis.
**18**	Case reports
**7**	Not accessible
**6**	Focusing on prevention
**5**	Focusing on fetus (clinical data and characteristics of the mother were missing or not complete)

4. Detailed distribution of patients on the studies included studies

Table S4. Detailed description about basal characteristics of included case-series.

Authors	Age	Gestation period (weeks)	Date of Diagnosis of COVID-19	Country	Place of residence	Symptoms at hospitalization	Obstetrical complications	SARS-Cov-2 quantitative RT-PCR	Postpartum fever (℃)	Respiratory symptoms	CT images	Treatment	Delivery method	Neonatal SARS-CoV-2 quantitative RT-PCR	Neonatal survival	Maternal survival
**Siyu Chen et al. (2020)[1)]**	29	40^{+4}	27/01/2020 14:30	China	Wuhan, Hubei	None	Gestational diabetes	-	38.5	-	GGO* in left lung	Antibiotics	Natural birth	-, Negative -	Alive	Alive
	30	39^{+1}	27/01/2020 09:50	China	Wuhan, Hubei	None	Preeclampsia	+	37.5	-	GGO in both lungs	Antibiotics	C-section	-, Negative	Alive	Alive
	25	38^{+6}	25/01/2020 23:43	China	Wuhan, Hubei	None	None	+	37.4	Cough	GGO in right lung	Antibiotics	Natural birth	-, Negative	Alive	Alive
	31	39^{+6}	28/01/2020 02:36	China	Wuhan, Hubei	None	None	+	37.4	-	GGO in right lung	Antibiotics	Natural birth	-, Negative	Alive	Alive
	29	39	28/01/2020 08:31	China	Wuhan, Hubei	None	Gestational diabetes	+	37.8	Sputum, coryza	GGO in both lungs	Antibiotics	C-section	-, Negative	Alive	Alive
**Liao J et al (2020)[2)]**	29	40^{+4}	20/01/2020~ 02/03/2020	China	Wuhan	Sore throat	-	Not done	-	Sore throat	Multiple patch GGO	-	Natural birth	Not done	Alive	Alive
	36	39	20/01/2020~ 02/03/2020	China	Wuhan	Cough	-	Not done	-	Cough	Multiple patch GGO	-	Natural birth	Not done	Alive	Alive
	30	39^{+5}	20/01/2020~ 02/03/2020	China	Wuhan	Fever	-	Not done	-	-	Multiple patch GGO	-	Natural birth	Not done	Alive	Alive
	36	37^{+6}	20/01/2020~ 02/03/2020	China	Wuhan	-	-	Not done	-	-	Multiple patch GGO	-	Natural birth	-, Negative	Alive	Alive
	36	38^{+1}	20/01/2020~ 02/03/2020	China	Wuhan	Fever	-	Not done	-	-	Multiple patch GGO	-	Natural birth	-, Negative	Alive	Alive
	30	40^{+1}	20/01/2020~ 02/03/2020	China	Wuhan	Cough	-	Not done	-	Cough	Multiple patch GGO	-	Natural birth	-, Negative	Alive	Alive
	33	39^{+4}	20/01/2020~ 02/03/2020	China	Wuhan	Cough	-	Not done	-	Cough	Multiple patch GGO	-	Natural birth	-, Negative	Alive	Alive
	33	36^{+2}	20/01/2020~ 02/03/2020	China	Wuhan	Fever	-	Not done	-	-	Multiple patch GGO	-	Natural birth	-, Negative	Alive	Alive
	27	40^{+2}	20/01/2020~ 02/03/2020	China	Wuhan	Fever, Chest pain	-	Not done	-	-	Multiple patch GGO	-	Natural birth	-, Negative	Alive	Alive
	29	37^{+2}	20/01/2020~ 02/03/2020	China	Wuhan	Fever	-	Not done	-	-	Multiple patch GGO	-	Natural birth	-, Negative	Alive, Hyaline membrane disease	Alive
**Luming Xu et al (2020)[3)]**	34	38^{+6}	21/01/2020~ 09/02/2020	China	Wuhan	Fever, Chest distress, Abdominal pain, anorexia	-	+	37.6	Chest distress	GGO in both lungs	Antibiotics, Antiviral	C-section	-, Negative	Alive	-
	25	34^{+4}	21/01/2020~ 09/02/2020	China	Wuhan	Fever	-	+	-	-	GGO in both lungs	Antibiotics, Antiviral, Corticosteroid	C-section	-, Negative	Alive	-

	23	37^{+3}	21/01/2020~ 09/02/2020	China	Wuhan	Fever,Chest distress ,Cou gh Dyspnea	-	+	38.7	Chest distress, Cough, Dyspnea	GGO in both lungs	Antibiotics Antiviral	C-section	-, Negative	Alive	-
	34	36^{+4}	21/01/2020~ 09/02/2020	China	Wuhan	Fever	-	+	-	-	GGO in both lungs	Antibiotics Antiviral, Corticosteroi d	C-section	-, Negative	Alive	-
	28	37	21/01/2020~ 09/02/2020	China	Wuhan	Fever ,Cough , Dyspnea, Abdominal pain	-	+	39.3	Cough	GGO in both lungs	Antibiotics Antiviral Corticosteroi d	Natural birth	-, Negative	Alive	-
**Buonsenso D et al (2020)[4]**	31	24	-	Italy	-	Fever, Cough, Shortness of breath	-	+	-	Cough, Shortness of breath	-	hydroxychlor oquine lopinavir/rito navir tolicizumab#, CPAP	-	Not done	Alive In pregnancy	Alive
	42	38	-	Italy	-	Fever, Cough	-	+	-	Cough	-	hydroxychlor oquine lopinavir/rito navir	C-section	-, Negative	Alive	Alive
	39	17	-	Italy	-	Cough, Ageusia, Anosmia	-	+	-	-	-	hydroxychlor oquine lopinavir/rito navir	-	Not done	Alive In pregnancy	Alive
	38	38	-	Italy	-	Fever,Cough	-	+	-	Cough	-	hydroxychlor oquine lopinavir/rito navir	C-section	-, Negative	Alive	Alive
**Dongmei CAO Et al (2020)[5]**	31	34^{+6}	-	China	Wuhan Hubei	Fever	Placental Abruption, Preeclampsia Premature	+	37.3-38.0	-	+, bilateral (abnl), Strip, pleural effusion	-	C-section	-, Negative	Alive premature	Alive
	29	40^{+3}	-	China	Wuhan Hubei	-	PROM, Fetal distress	+	-	-	+, single, GGO	-	C-section	Not done	Alive	Alive
	30	38^{+1}	-	China	Wuhan Hubei	-	-	+	-	-	+, single, Patch	-	Natural birth	Not done	Alive	Alive
	35	33^{+6}	-	China	Wuhan Hubei	Fever	Premature, PROM	+	37.3-38.0	-	+, bilateral, patch,Pleural thickening	-	C-section	-, Negative	Alive	Alive
	29	40^{+0}	-	China	Wuhan Hubei	Fever	Fetal distress	+	38.1-39.0	-	+, bilateral,patch, Pleural effusion	-	C-section	-, Negative	Alive	Alive
	31	38^{+3}	-	China	Wuhan Hubei	Fever	PROM, Preeclampsia	+	38.1-39.0	-	+, bilateral,patch, pleural effusion	-	C-section	Not done	Alive	Alive
	30	35^{+0}	-	China	Wuhan Hubei	-	Premature, PROM, twin	+	-	-	+,single, strip, GGO	-	C-section	-, Negative	Alive	Alive

	29	39^{+2}	-	China	Wuhan Hubei	Fever	Hypothyroidism, Anemia	+	>39.1	-	+, bilateral, pleural effusion	-	C-section	Not done	Alive	Alive
	30	39^{+1}	-	China	Wuhan Hubei	Fever	Preeclampsia	+	37.3-38.0	-	+ , bilateral, patch, GGO, pleural effusion	-	C-section	Not done	Alive	Alive
	29	40^{+6}	-	China	Wuhan Hubei	Fever, Cough, Chest tightness, Fatigue	GDM	+	38.1-39.0	Cough, Chest tightness	+, single, patch, GGO	-	Natural birth	Not done	Alive	Alive
**Chunchen Wu Et al(2020)[6]**	28	39	-	China	Wuhan Hubei	Fever	-	+	-	-	-	antibiotics	C-section	No result	-	-
	31	38^{+1}	-	China	Wuhan Hubei	Fever	PROM	+	-	-	-	Antibiotics	C-section	No result	-	-
	30	39^{+1}	-	China	Wuhan Hubei	-	Preeclampsia	+	-	-	GGO, small plaque	Antibiotics	C-section	No result	-	-
	30	36^{+4}	-	China	Wuhan Hubei	-	PROM	+	-	-	GGO, small plaque	Antibiotics	C-section	No result	-	-
	30	37^{+6}	-	China	Wuhan Hubei	-	-	+	-	-	GGO, small plaque	Antibiotics	Natural birth	No result	-	-
	26	40^{+3}	-	China	Wuhan Hubei	-	Fetal distress	+	-	-	GGO, small plaque	Antibiotics	C-section	No result	-	-
	29	40^{+4}	-	China	Wuhan Hubei	Fever	-	Not done	-	-	GGO, small plaque	Antibiotics	Natural Birth	No result	-	-
	35	33^{+6}	-	China	Wuhan Hubei	Fever	-	Not done	-	-	GGO, small plaque	antibiotics	C-section	No result	-	-
**Pu Yang Et al (2020)[7]**	-	37^{+2}	33^{+6}	China	Wuhan	Fever	-	+	-	-	+, viral pneumonia	-	C-section	-,Negative	Alive	-
	-	38^{+2}	23/01/2020	China	Wuhan	Fever	-	+	-	-	+	-	C-section	-,Negative	Alive	-
	-	36^{+2}	27/01/2020	China	Wuhan	Fever, diarrhea	-	+	-	-	+	-	C-section	-,Negative	Alive	-
	-	36^{+3}	28/01/2020	China	Wuhan	Cough	-	+	-	Cough	+	-	C-section	-,Negative	Alive	-
	-	36	29/01/2020	China	Wuhan	Abdominal pain	-	+	-	-	+	-	C-section	-,Negative	Alive	-
	-	36^{+2}	29/01/2020	China	Wuhan	Fever	-	+	-	-	Normal	-	C-section	No result	Alive	-
	-	38	27/01/2020	China	Wuhan	Fever	-	+	-	-	+	-	C-section	-,Negative	Alive	-
**Xiaoqing Et al (2020)[8]**	27	39	-	China	Wuhan	-	-	-, negative	-	-	-	-	-	Not done	-	-
	29	37^{+6}	-	China	Wuhan	Fever, Cough	-	+	-	Cough	Multiple patchy, wedge shaped GGO	-	-	Not done	-	-
	28	39^{+1}	-	China	Wuhan	-	Threatened abortion	-, negative	-	-	-	-	-	-, negative	-	-

	26	38^{+5}	-	China	Wuhan	Nasal obstruction	-	+	-	Nasal obstruction	-	-	-	Not done	-	-
	33	38^{+6}	-	China	Wuhan	-	Threatened abortion	-, negative	-	-	Focal GGO , Rt. lobe	-	-	Not done	-	-
	28	38^{+5}	-	China	Wuhan	Cough	-	+	-	Cough	Pathy consolidation Fibrous stripes	-	-	Not done	-	-
	34	38^{+3}	-	China	Wuhan	-	PROM	+	-	-	-	-	-	-, negative	-	-
	28	39^{+1}	-	China	Wuhan	-	-	+	-	-	GGO, bilateral In lower lobes	-	-	Not done	-	-
	24	12	-	China	Wuhan	-	Threatened abortion Pregnancy induced HTN	+	-	-	-	-	-	Not done	-	-
	32	38	-	China	Wuhan	Cough	-	+	-	Cough	GGO, Rt lobe	-	-	Not done	-	-
	37	6	-	China	Wuhan	-	PROM Pregnancy Induced HTN	-, negative	-	-	-	-	-	Not done	-	-
	22	8	-	China	Wuhan	Fever, Cough	-	+	-	Cough	-	-	-	Not done	-	-
	30	35^{+4}	-	China	Wuhan	-	Pregnancy Induced HTN	+	-	-	GGO, Rt. lobe	-	-	Not done	-	-
	26	37^{+3}	-	China	Wuhan	Fever, Cough	-	+	-	Cough	-	-	-	-, negative	-	-
	29	39	-	China	Wuhan	-	-	+	-	-	-	-	-	Not done	-	-
	21	39^{+9}	-	China	Wuhan	-	-	+	-	-	-	-	-	-, negative	-	-
	26	38	-	China	Wuhan	-	-	+	-	-	-	-	-	Not done	-	-
	32	31^{+5}	-	China	Wuhan	-	-	+	-	-	-	-	-	Not done	-	-
	35	37^{+6}	-	China	Wuhan	Fever	-	+	-	-	-	-	-	Not done	-	-
	30	40	-	China	Wuhan	-	Fetal intrauterine hypoxia	+	-	-	-	-	-	Not done	-	-
	36	38^{+1}	-	China	Wuhan	-	Pregnancy Induced HTN	+	-	-	-	-	-	Not done	-	-
	30	31^{+8}	-	China	Wuhan	-	-	+	-	-	-	-	-	Not done	-	-
	29	37^{+2}	-	China	Wuhan	-	-	+	-	-	-	-	-	Not done	-	-
**Liao J et al (2020)**[9]	25	-	-	China	Wuhan Hubei	Fever	Intrauterine fetal distress Cord torsion Oligohydramnio s	+	-	-	개별 결과 Not reported	-	C-section	-, negative	-	-

	35	-	-	China	Wuhan Hubei	Fever	Scarred uterus PROM	+	-	-	Not reported	-	C-section	-, negative	-	-
	35	-	-	China	Wuhan Hubei	Cough	Intrauterine fetal distress PROM	+	-	Cough	Not reported	-	Natural birth	-, negative	-	-
	30	-	-	China	Wuhan Hubei	Fever	Intrauterine fetal distress Vaginal bleeding In the 3rd trimester	+	-	-	Not reported	-	C-section	-, negative	-	-
	30	-	-	China	Wuhan Hubei	Cholecystitis, Fever	-	+	-	-	Not reported	-	C-section	Not done	-	-
	30	-	-	China	Wuhan Hubei	Fever, Cough	Intrauterine fetal distress Cord-colloid Placenta previa Polyhydramnios	+	-	Cough	Not reported	-	C-section	-, negative	-	-
	30	-	-	China	Wuhan Hubei	Fever, Cough, sore Throat, diarrhea	Intrauterine fetal distress PROM	+	-	Cough, Sore throat	Not reported	-	C-section	-, negative	-	-
	29, twin(1)	-	-	China	Wuhan Hubei	Fever, Cough	Intrauterine fetal distress	-, negative	-	Cough	Bilateral GGO, patchy consolidation, blurred border	-	Natural birth	-, negative	-	-
	29, Twin(2)	-	-	China	Wuhan Hubei	Fever, Cough	Intrauterine fetal distress	-,negative	-	Cough	Bilateral GGO, patchy consolidation, blurred border	-	Natural birth	-, negative	-	-
	34	-	-	China	Wuhan Hubei	Fever	-	+	-	-	Not reported	-	C-section	-, negative	-	-
**Yangli Liu et al (2020)**[10]	28	25	-	China	-	Fever, Fatigue	-	Not reported	-		-	-	NA	Not reported	Alive	Alive
	24	27	-	China	-	Fever, Dyspnea	-	Not reported	38.5	Dyspnea	-	-	NA	Not reported	Alive	Alive
	33	32	-	China	-	Cough	Prematurity	Not reported	-	Cough	-	-	C-section	Not reported	Alive	Alive
	29	33	-	China	-	Fever	-	Not reported	38	-	-	-	NA	Not reported	Alive	Alive
	35	34	-	China	-	Fever	PROM, prematurity	Not reported	-	-	-	-	C-section	Not reported	Alive	Alive
	31	34	-	China	-	Fever, sore throat	MODS Stillbirth Prematurity	Not reported	-	Sore throat	-	-	C-section	Not reported	Death	Alive
	30	35	-	China	-	Dyspnea	Fetal distress Prematurity	Not reported	-	Dyspnea	-	-	C-section	Not reported	Alive	Alive
	36	35^{+5}	-	China	-	Fever, cough, dyspnea	Prematurity	Not reported	-	Cough, dyspnea	-	-	C-section	Not reported	Alive	Alive
	26	36	-	China	-	Fever, fatigue	Prematurity	Not	-	-	-	-	C-section	Not reported	Alive	Alive

								reported							
	32	36^{+4}	-	China	-	Fever, fatigue	Fetal distress	Not reported	39	-	-	-	C-section	Not reported	Alive	Alive
	30	37	-	China	-	Fever, fatigue	Fetal distress	Not reported	-	-	-	-	C-section	Not reported	Alive	Alive
	22	38	-	China	-	-	-	Not reported	-	-	-	-	C-section	Not reported	Alive	Alive
	30	38^{+3}	-	China	-	Fever	-	Not reported	37.3	-	-	-	C-section	Not reported	Alive	Alive
**Sedigheh Hantoushzadeh et al (2020)[11]**	25-29	30^{+3}	-	Iran	-	Fever,Cough Dyspnea, Myalgia Sore throat Rhinorrhea	Fetal death	+	-	Cough Dyspnea	+	Antibiotics Antiviral	Natural birth	Not done	Death	Death
	25-29	38^{+3}	-	Iran	-	Fever,Cough Myalgia, dyspnea	-	+	-	Cough Dyspnea	Bilateral patchy	Antibiotics Antiviral, Anticoagulant	C-section	-, negative	Alive	Death
	40-44	30^{+5}	-	Iran	-	Fever,Cough Dyspnea	-	+	-	Cough Dyspnea	Bilateral Patchy GGO	Antibiotics Antiviral, Anticoagulant HCQ	C-section	-, negative	Alive	Death
	30-34	24^{+7}	-	Iran	-	Fever,Cough Dyspnea, Myalgia	Fetal death	+	-	Cough Dyspnea,	Bilateral patch GGO	Antibiotics Antiviral, Anticoagulant HCQ	NA-	Not done	Death	Death
	30-34	36	-	Iran	-	Fever,Cough dyspnea	-	+	-	Cough Dyspnea ,	-	Antibiotics Antiviral, Anticoagulant	C-section	-, negative-	Alive	Death
	35-39	24	-	Iran	-	Fever,Cough Dyspnea, Myalgia	Fetal death	+	-	Cough Dyspnea	Bilateral patchy GGO	Antibiotics Antiviral, Anticoagulant HCQ,IVIG	NA	Not done	Death (twin DCDA)	Death
	45-49	28	-	Iran	-	Fever,Cough Dyspnea	-	+	-	Cough Dyspnea	Bilateral patchy GGO	Antibiotics Antiviral, Anticoagulant HCQ	C-section	-, negative	Death after birth (twin DCDA)	Death
	35-39	33^{+5}	-	Iran	-	Fever,Cough Dyspnea, Hemoptysis	-	+	-	Cough Dyspnea hemoptysis	Bilateral patchy GGO	Antibiotics Antiviral, Anticoagulant HcQ,Plasma	C-section	-, negative	Alive	Alive-
	35-39	36	-	Iran	-	Fever,Cough Dyspnea	Fetal death PPROM	+	-	Cough Dyspnea	Bilatral PatchyGGO	Antibiotics Antiviral, Anticoagulant	C-section	Not done	Death	Alive-

HcQ

GGO : ground glass opacity; DCDA; dichorionic, diamniotic twin gestation, COVID; coronavirus disease 2019, HCQ; hydroxychloroquine, PPROM; preterm premature rupture of membrane, PROM; premature rupture of membrane, HTN; hypertension, NA; not available, C-section; caesarean section, Rt; right.

5. Reference

1. Chen S, Liao E, Cao D, Gao Y, Sun G, Shao Y. Clinical analysis of pregnant women with 2019 novel coronavirus pneumonia. J Med Virol 2020;
2. Liao J, He X, Gong Q, Yang L, Zhou C, Li J. Analysis of vaginal delivery outcomes among pregnant women in Wuhan, China during the COVID-19 pandemic. Int J Gynaecol Obstet 2020;
3. Xu L, Yang Q, Shi H, Lei S, Liu X, Zhu Y, et al. Clinical presentations and outcomes of SARS-CoV-2 infected pneumonia in pregnant women and health status of their neonates. Sci Bull (Beijing) 2020;
4. Buonsenso D, Raffaelli F, Tamburrini E, Biasucci DG, Salvi S, Smargiassi A, et al. Clinical role of lung ultrasound for the diagnosis and monitoring of COVID-19 pneumonia in pregnant women. Ultrasound Obstet Gynecol 2020;
5. Cao D, Yin H, Chen J, Tang F, Peng M, Li R, et al. Clinical analysis of ten pregnant women with COVID-19 in Wuhan, China: A retrospective study. Int J Infect Dis 2020;
6. Wu C, Yang W, Wu X, Zhang T, Zhao Y, Ren W, et al. Clinical Manifestation and Laboratory Characteristics of SARS-CoV-2 Infection in Pregnant Women. Virol Sin 2020;
7. Yang P, Wang X, Liu P, Wei C, He B, Zheng J, et al. Clinical characteristics and risk assessment of newborns born to mothers with COVID-19. J Clin Virol 2020;127:104356.
8. Wu X, Sun R, Chen J, Xie Y, Zhang S, Wang X. Radiological findings and clinical characteristics of pregnant women with COVID-19 pneumonia. Int J Gynaecol Obstet 2020;
9. Zhu H, Wang L, Fang C, Peng S, Zhang L, Chang G, et al. Clinical analysis of 10 neonates born to mothers with 2019-nCoV pneumonia. Transl Pediatr 2020;9:51-60.
10. Liu Y, Chen H, Tang K, Guo Y. Clinical manifestations and outcome of SARS-CoV-2 infection during pregnancy. J Infect 2020;
11. Hantoushzadeh S, Shamshirsaz AA, Aleyasin A, Seferovic MD, Aski SK, Arian SE, et al. Maternal Death Due to COVID-19 Disease. Am J Obstet Gynecol 2020;
